# WFDC3 identified as a prognostic and immune biomarker in pancreatic cancer

**DOI:** 10.17305/bb.2025.12444

**Published:** 2025-05-08

**Authors:** Bohan Liu, Xuqing Shi, Tianqi Liu, Huanwen Wu, Zhiyong Liang

**Affiliations:** 1 Molecular Pathology Research Center, Department of Pathology, Peking Union Medical College Hospital, Chinese Academy of Medical Sciences and Peking Union Medical College, Beijing, China; 2 Department of Pathology, State Key Laboratory of Complex Severe and Rare Disease, Molecular Pathology Research Center, Peking Union Medical College Hospital, Chinese Academy of Medical Sciences and Peking Union Medical College, Beijing, China

**Keywords:** Pancreatic cancer, whey acidic protein four-disulfide core, WFDC3, bioinformatics, metastasis, immunotherapy, prognosis

## Abstract

The whey acidic protein four-disulfide core (WFDC) family comprises key modulators of tumor initiation and progression, offering significant potential for diagnostic, prognostic, and therapeutic applications. However, the specific role of WFDCs in the oncogenesis of pancreatic cancer (pancreatic adenocarcinoma [PAAD]) remains incompletely understood. To address this, we conducted an initial investigation using comprehensive bioinformatic analyses to evaluate *WFDCs* expression patterns across multiple tumor types, with a focus on PAAD. Bulk and single-cell RNA sequencing datasets from the TCGA and GEO repositories were analyzed to assess *WFDC3* expression in PAAD tissues. Kaplan–Meier survival analysis was employed to determine the prognostic significance of *WFDC3*. To explore its biological functions and underlying mechanisms, we performed functional enrichment analyses in combination with immune infiltration assessments. Experimental validation included CCK-8 and EdU proliferation assays, transwell migration and invasion tests, immunofluorescence staining, flow cytometry, LDH release assays, Western blotting, and quantitative reverse transcription PCR. A LASSO regression model was also developed to predict PAAD outcomes. Our findings reveal that WFDCs exhibit context-dependent roles in tumor progression. Specifically, *WFDC3* expression was significantly elevated in PAAD and associated with poorer patient prognosis. Functionally, WFDC3 promoted PAAD cell metastasis by inducing epithelial–mesenchymal transition and contributed to immune evasion by suppressing T cell cytotoxicity. In conclusion, our study identifies WFDC3 as a pro-oncogenic factor in PAAD progression, highlighting its potential as both a prognostic biomarker and a therapeutic target for regulating metastasis and immune responses in this malignancy.

## Introduction

Pancreatic cancer (pancreatic adenocarcinoma [PAAD]) is an aggressive malignancy that presents significant challenges in both diagnosis and treatment, primarily due to its insidious onset, high metastatic potential, and the limited efficacy of current therapeutic strategies [1]. Epidemiological data show that PAAD has nearly identical incidence and mortality rates, with a dismal five-year survival rate of approximately 10% [2, 3]. This clinical reality underscores the urgent need for novel biomarkers capable of predicting outcomes, monitoring recurrence, and identifying therapeutic targets. Although recent advances in immunotherapy have shown promising efficacy in various cancer types, their clinical benefit for PAAD patients remains substantially limited [4]. Evidence suggests that the pancreatic tumor microenvironment is enriched with immunosuppressive cells and factors that collectively impair effective immune responses [5]. Therefore, characterizing this immunosuppressive microenvironment is crucial for identifying new immunotherapeutic targets in PAAD. The whey acidic protein four-disulfide core (WFDC) family comprises 18 distinct genes, 14 of which are located on human chromosome 20. Most members encode secretory proteins of relatively small molecular weight [6]. These proteins are defined by the presence of one or more WFDC domains, featuring conserved structural motifs of 40–50 amino acids, within which eight cysteine residues form four disulfide bridges through specific bonding patterns [7, 8]. WFDC proteins are broadly expressed across human tissues—particularly in the reproductive and respiratory systems—where they participate in diverse biological processes, including protease inhibition, antimicrobial activity, and immune modulation [9]. Dysregulated WFDC expression has been significantly associated with various pathological conditions, especially in the development and metastatic progression of several malignancies [10–12], highlighting the need for systematic, multi-omics studies of WFDCs in pan-cancer contexts. WFDCs exhibit cancer type-dependent functional diversity, reflecting their heterogeneous biological roles in malignant conditions. Elevated serum levels of WFDC2 have been validated as clinically relevant biomarkers for diagnostic evaluation and prognostic stratification in lung cancer [13]. In ovarian carcinoma, WFDC2 has emerged as a biomarker with high diagnostic accuracy across multiple clinical studies [14]. Conversely, prostate cancer specimens often show significant downregulation of WFDC2, correlating inversely with Gleason grade parameters [15]. Previous research from our group identified a tumor-suppressive role of WFDC3 in inhibiting estrogen-dependent metastasis via activation of the ERβ/TGFBR1 pathway [16]. Additionally, we found that WFDC3 serves as a predictive marker for enhanced chemotherapeutic response in colorectal cancer (CRC), through modulation of the ATM/ATR kinase signaling pathways [17]. Nevertheless, the pathophysiological relevance of WFDC3 in other malignancies—particularly PAAD—remains insufficiently explored and warrants further mechanistic investigation.

In this study, we conducted a comprehensive pan-cancer analysis to evaluate the expression patterns of *WFDCs* and their correlations with the immune microenvironment across 33 malignancies, using bulk RNA-seq datasets from the TCGA and GTEx repositories. We then focused specifically on *WFDC3* expression in PAAD, utilizing both bulk transcriptomic and single-cell RNA sequencing (scRNA-seq) data to elucidate associated molecular pathways. Through a combination of computational analyses and experimental validation, we found that *WFDC3* expression was significantly elevated in PAAD specimens and that higher expression levels were strongly associated with poor clinical outcomes. Notably, our study is the first to demonstrate that *WFDC3* knockdown markedly suppresses tumor cell invasion and migration while enhancing susceptibility to immune-mediated cytotoxicity. Finally, we developed a *WFDCs*-based prognostic signature to stratify survival outcomes in PAAD patients. Together, these findings highlight the dual role of WFDCs as both prognostic biomarkers and promising therapeutic targets in PAAD management.

## Materials and methods

### Genomic data and clinical information

Transcriptomic profiles, clinicopathological parameters, and somatic mutation datasets for 33 solid tumors were obtained from the TCGA database (https://portal.gdc.cancer.gov/) and the UCSC Xena platform (https://xena.ucsc.edu/). Corresponding normal tissue transcriptomic data were sourced from the GTEx project (https://commonfund.nih.gov/GTEx) for comparative analysis. FPKM values were normalized to TPM to enable unbiased comparisons. To correct for potential batch effects arising from the integration of TCGA and GTEx datasets, we applied the ComBat_seq function from the “sva” R package, which adjusts for known batch variables in RNA-seq count data while preserving biological signals. Data preprocessing included the exclusion of non-compliant records with missing entries or duplicates, followed by data visualization using the “ggplot2” package (v3.4.4) in the R programming environment.

### Expression and prognostic analysis

The TIMER platform (https://cistrome.shinyapps.io/timer/) and the GEPIA database (http://gepia.cancer-pku.cn/) were utilized as primary analytical tools to investigate *WFDCs* expression patterns across various cancer types. Prognostic evaluation of *WFDCs* in pan-cancer contexts was conducted using the GSCA resource (https://guolab.wchscu.cn/GSCA). TCGA and GTEx datasets were subsequently used to validate the expression characteristics of *WFDCs* specifically in PAAD. The CRC immunotherapy cohort GSE53127 was obtained from the GEO database for further analysis. Kaplan–Meier survival curves were generated using the “survival” (v3.3.1) and “survminer” (v0.4.9) R packages to illustrate the associations of *WFDCs* with overall survival (OS), disease-specific survival (DSS), and progression-free interval (PFI) in PAAD. Additionally, the Kaplan–Meier method combined with univariate Cox regression analysis was applied to assess the prognostic significance of *WFDC3* in relation to OS, DSS, and PFI across multiple cancer types.

### scRNA-seq analysis

Initial investigation of *WFDC3* expression patterns at single-cell resolution across diverse cell types in PAAD was conducted using the TISCH database (http://tisch.comp-genomics.org/). Transcriptomic data for *WFDC3* mRNA levels were obtained from the GSE154778 dataset in the GEO repository (https://www.ncbi.nlm.nih.gov/geo/), and subsequently processed using Python 3.12.2 with Scanpy (v1.10.2) [18]. Dimensionality reduction was performed via principal component analysis, and batch effect correction was applied using the “scvi-tools” Python package (v1.2.0) [19, 20]. Cellular subpopulations were identified using the Leiden clustering algorithm on UMAP-transformed data, with cell type annotation based on known marker genes. Malignant cells were distinguished through chromosomal instability profiling using the “infercnvpy” Python package (v0.5.0) for single-cell CNV analysis. The spatial distribution and expression intensity of *WFDC3* were visualized using UMAP projections and violin plots. Independent validation was conducted by re-analyzing the original scRNA-seq dataset with the Seurat analytical pipeline.

### Clinicopathological and mutational analysis

Clinical and pathological features, along with somatic mutation profiles for 178 PAAD patients, were obtained from the TCGA-PAAD cohort. The associations between these features, genomic mutations, and *WFDC3* expression were subsequently evaluated using the Mann–Whitney *U* test.

### Identification of differentially expressed genes (DEGs) and functional enrichment analysis

To investigate biological processes and signaling pathways associated with *WFDC3* expression, differential mRNA analysis was first conducted between *WFDC3*-low and *WFDC3*-high subgroups using TCGA-PAAD transcriptomic datasets via the “DESeq2” R package (v1.36.0). Significantly dysregulated genes were visualized using MA plots, applying predefined thresholds of adjusted *P* value (p.adj) < 0.05 and absolute log_2_ fold change (|log2FC|) > 1. Functional enrichment analyses were subsequently performed using the “ClusterProfiler” R package (v4.4.4) to identify enriched Gene Ontology (GO) terms and Kyoto Encyclopedia of Genes and Genomes (KEGG) pathways, with statistical significance set at p.adj < 0.05. To compare signaling pathway differences between WFDC3 expression subgroups, Gene Set Enrichment Analysis (GSEA) was conducted using hallmark gene sets (h.all.v7.5.1.symbols.gmt) and immunologic signature collections (c7.all.v2022.1.Hs.symbols.gmt).

### Tumor microenvironment and immune infiltration analysis

Immune infiltration scores for TCGA-PAAD cohort samples were calculated using the CIBERSORT computational method [21]. Comparative analysis revealed differences in immune cell composition between PAAD patients stratified by *WFDC3* expression levels, with results visualized using the “ggplot2” package. Stromal and immune cell proportions within the PAAD tumor microenvironment were estimated using the ESTIMATE algorithm to assess tumor purity [22]. To explore the relationship between *WFDC3* expression and T lymphocyte subpopulations, ssGSEA was performed using the “GSVA” R package (v1.46.0) [23, 24].

### Development and validation of a prognostic model based on *WFDCs*

A LASSO regression approach was implemented using the “glmnet” R package (v4.1.7) to evaluate 18 WFDC family members, with the optimal regularization parameter (λ) determined through cross-validation. Model construction utilized ten-fold cross-validation to ensure robustness and predictive generalizability. Four WFDC members (*WFDC3*, *WFIKKN1*, *SLPI*, *PI3*) showing significant associations with OS were incorporated into the prognostic signature. Risk stratification was based on the following formula: Risk Score ═ (0.068 × *WFDC3*) + (−0.5398 × *WFIKKN1*) + (0.041 × *PI3*) + (0.0738 × *SLPI*), enabling classification of patients into low- and high-risk groups using the median risk score as a threshold. Model validation included Kaplan–Meier survival curves, ROC analysis, calibration plots, univariate forest plots, and prognostic nomogram construction to assess clinical predictive accuracy in PAAD. To explore immune landscape differences, Spearman’s rank correlation analysis was performed to compare immune cell infiltration patterns between risk groups. To strengthen the prognostic model’s credibility, external validation was conducted using two independent PAAD cohorts from the GEO database (GSE62452 and GSE78229). Expression levels of the four genes were normalized, and individual risk scores were calculated using the established formula. Patients were stratified into high- and low-risk groups based on the median score, and survival differences were assessed via Kaplan–Meier analysis.

### Cell culture and cell transfection

The human pancreatic ductal epithelial cell line HPNE, the human T cell line Jurkat, and the human pancreatic cancer cell lines MIA PaCa-2, PANC-1, AsPC-1, and BxPC-3 were obtained from the American Type Culture Collection (ATCC). MIA PaCa-2 and PANC-1 cells were cultured in Dulbecco’s Modified Eagle’s Medium (DMEM; Thermo Fisher Scientific, Waltham, MA, USA), while AsPC-1, BxPC-3, and Jurkat T cells were maintained in Roswell Park Memorial Institute Medium 1640 (RPMI-1640; Thermo Fisher Scientific, Waltham, MA, USA). All culture media were supplemented with 10% fetal bovine serum (FBS). HPNE cells were cultured in a customized medium consisting of 75% low-glucose DMEM and 25% Medium M3 Base (both from Thermo Fisher Scientific), supplemented with 5% FBS, 10 ng/mL human recombinant epidermal growth factor (EGF; PeproTech, NJ, USA), and 750 ng/mL puromycin. All cell lines were maintained at 37 ^∘^C in a humidified atmosphere containing 5% CO_2_, with regular medium replacement and passaging according to standard protocols. For gain- and loss-of-function experiments, a *WFDC3* overexpression plasmid (GeneChem Co., Ltd., China) was transfected into PANC-1 cells using Lipofectamine 3000 reagent (Invitrogen, Catalog No. 3000155) at 80%–90% confluency. For gene knockdown, ON-TARGETplus SMARTpool *WFDC3* siRNA (Dharmacon, Catalog No. L-003402-00-0005, USA), a mixture of four siRNAs targeting *WFDC3*, was transfected into MIA PaCa-2 cells during the logarithmic growth phase using Lipofectamine RNAiMAX reagent (Invitrogen, Catalog No. 13778) at 70%–80% confluency, following the manufacturer’s protocol.

### Enzyme-linked immunosorbent assay (ELISA)

WFDC3 protein levels in conditioned medium were quantified using a human-specific ELISA kit (Yuanju Biotechnology Co., Ltd., cat. YJ290758, Shanghai, China). Standard solutions, serum samples, and HRP-conjugated antigens were added to pre-coated assay plates according to the manufacturer’s instructions. After a 30-min incubation at 37 ^∘^C, residual liquid was discarded, and the plates were washed five times. Then, 50 µL of TMB substrate was added and incubated in the dark at 37 ^∘^C for 10 min. The reaction was stopped by adding 50 µL of stop buffer, and absorbance was measured immediately at 450 nm.

### Cell viability and proliferation assay

Multiple experimental approaches were used to assess the proliferative capacity of PANC-1 cells following transfection with the *WFDC3* overexpression plasmid. Cell viability was measured at defined time points using the Cell Counting Kit-8 (Dojindo, Kumamoto, Japan). Colony formation was analyzed by fixing and staining cells with 0.5% crystal violet, followed by digital image capture. EdU incorporation assays were performed using the Cell-Light EdU Apollo643 *In Vitro* Kit (Ribobio, #C10310-2), according to the manufacturer’s protocol. Fluorescent signals were then acquired and quantified using High Content Analysis (HCA) instrumentation.

### Transwell migration and invasion assay

Transwell chambers were employed for migration and invasion assays. For the invasion assay, the upper surfaces of the Transwell inserts were uniformly coated with extracellular Matrigel diluted 1:10. Differentially treated PAAD cells (5 × 10^4^), suspended in 100 µL of serum-free medium, were seeded into the coated upper chambers. The lower chambers of 24-well plates were filled with 500 µL of complete medium containing 10% FBS and incubated at 37 ^∘^C with 5% CO_2_ for 24–48 h. Cells were fixed with 4% paraformaldehyde, and invasion was quantified using crystal violet staining. Membranes were imaged via bright-field microscopy, and cell counts were performed using ImageJ software.

### Immunofluorescence (IF) staining

Cells were seeded onto glass coverslips and subjected to experimental treatments. After fixation, the slides were blocked with goat serum and incubated overnight at 4 ^∘^C with primary antibodies: E-cadherin (1:200, #14472S; CST, USA), N-cadherin (1:1000, #14215; CST, USA), and ZO-1 (1:200, #13663; CST, USA). Following washes, slides were incubated at room temperature for 30 min with species-matched secondary antibodies conjugated to FITC (ZF-0312) or TRITC (ZF-0316) (1:1000; ZSGB-BIO), then counterstained with DAPI (Beyotime) for nuclear visualization. Slides were mounted using ProLong™ Glass Antifade Mountant (Invitrogen), and images were acquired using a Nikon AXR laser scanning confocal microscope.

### Flow cytometry

Jurkat T lymphocytes were subjected to experimental treatments, followed by a 6-h exposure to a cell activation cocktail containing Brefeldin A (BioLegend, 423303) to facilitate intracellular cytokine detection. After fixation and membrane permeabilization, intracellular Granzyme B (GZMB) was stained using an APC-conjugated anti-GZMB monoclonal antibody (BioLegend, 372203). Fluorescence signals were analyzed by flow cytometry, and quantitative data were processed using FlowJo software (v10.8.1).

### LDH release assay

*WFDC3*-knockdown MIA PaCa-2 cells (2 × 10^3^) or *WFDC3*-overexpressing PANC-1 cells (2 × 10^3^) were co-cultured with Jurkat T cells (1 × 10^4^) in 96-well plates with opaque walls and flat bottoms, using complete tumor cell medium (DMEM), for 48 h at an optimal effector-to-target (E:T) ratio of 5:1. Tumor cells and Jurkat T cells cultured separately served as controls to establish baseline death rates. Cell death was measured using the LDH-Glo™ Cytotoxicity Assay kit (Promega, Catalog No. J2380), and death rates were calculated according to the manufacturer’s protocol.

### Quantitative real-time PCR (qRT-PCR)

RNA was extracted using the RNA-Quick Purification Kit (Yishan Biotechnology Co., Ltd., cat. RNOOl, Shanghai, China), and reverse transcription into cDNA was carried out with PrimeScript™ RT Master Mix (TaKaRa, Japan) according to the manufacturer’s instructions. qRT-PCR was then performed using the QuantiNova SYBR PCR Mix Kit (cat. 4993626, QIAGEN, Germany). GAPDH was used as the endogenous reference gene for normalization. The nucleotide sequences of the amplification primers are listed in Table S1.

### Western blot analysis

Cellular lysates were prepared using RIPA buffer supplemented with a protease and phosphatase inhibitor cocktail (Roche Applied Science, Indianapolis, IN, USA). Protein concentrations were determined using the Pierce BCA Protein Assay Kit (Thermo Fisher Scientific). Proteins were separated by electrophoresis and transferred onto polyvinylidene difluoride (PVDF) membranes (Millipore, Billerica, MA, USA). Membranes were blocked for 1 h at room temperature with 5% nonfat milk, followed by overnight incubation at 4 ^∘^C with primary antibodies. The next day, membranes were incubated with secondary antibodies, and signal detection was performed using the ChemiDoc Touch Imaging System (Bio-Rad). GAPDH was used as a loading control. Detailed information on the antibodies used for Western blotting is provided in Table S2. Uncropped Western blot images with clearly labeled molecular weight (kDa) markers are included in the supplementary file.

### Statistical analysis

Statistical analyses were conducted using R v4.2.1, Python v3.12.2, ImageJ, and GraphPad Prism v10.2.1. Experimental data from triplicate trials are presented as mean ± SEM. This level of replication was selected in accordance with established standards in molecular biology research to balance statistical rigor with practical constraints on time and resources. Comparisons between two independent groups were assessed using either Student’s *t*-test or the Wilcoxon rank-sum test, while multi-group comparisons employed one-way ANOVA followed by Kruskal–Wallis post hoc testing. Survival outcomes, including OS, DSS, and PFI, were evaluated using the Kaplan–Meier method, with survival curves used for graphical representation. For non-normally distributed quantitative variables, correlations were assessed using Pearson’s correlation coefficient. Statistical significance was determined using the log-rank test, with a threshold of *P* < 0.05.

**Figure 1.**
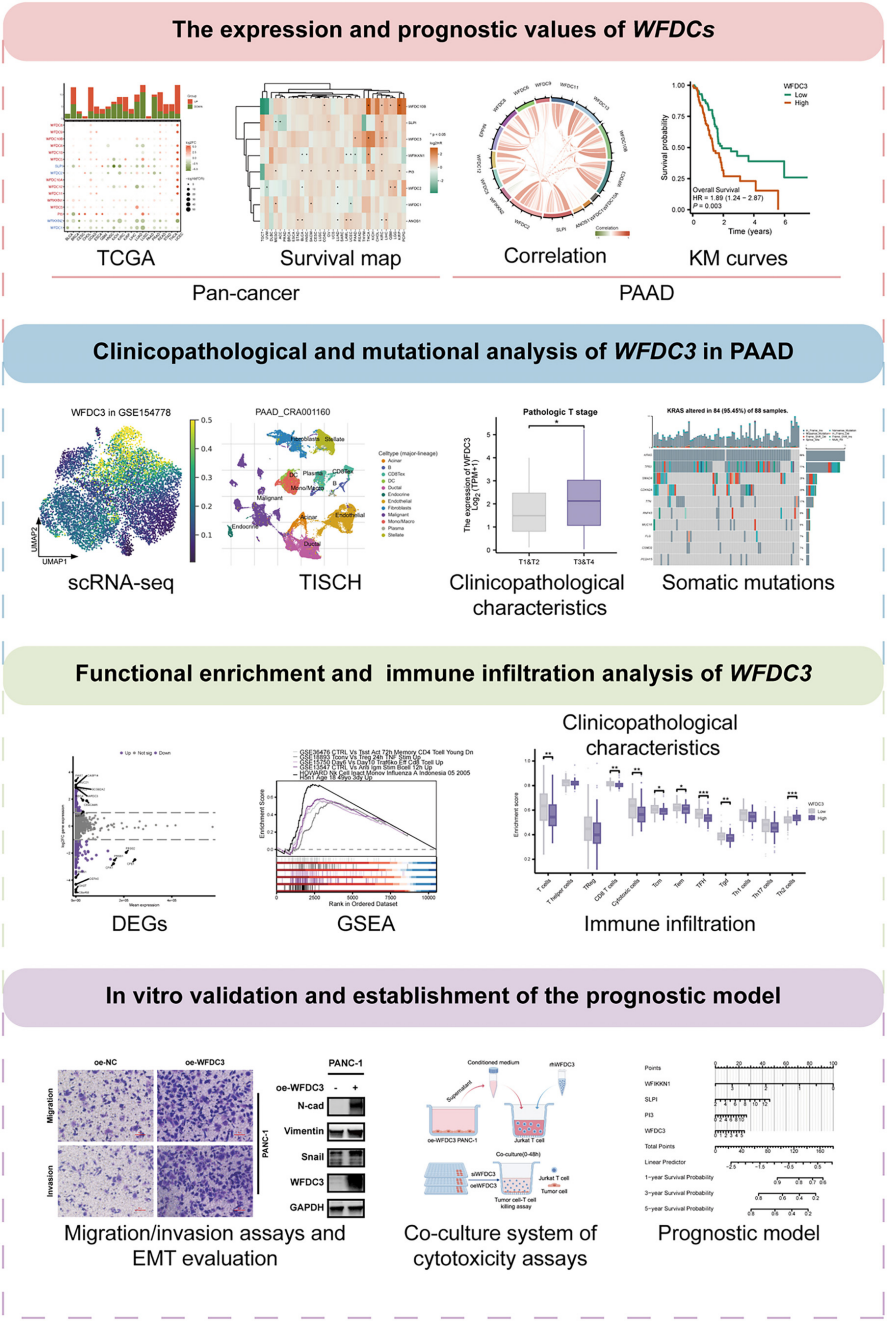
**The flowchart of this study.** DEG: Differentially expressed gene; EMT: Epithelial–mesenchymal transition; GSEA: Gene Set Enrichment Analysis; scRNA-seq: Single-cell RNA sequencing; WFDC: Whey acidic protein four-disulfide core.

## Results

### Landscape of WFDC family gene expression and prognostic significance in pan-cancer

**Figure 2.**
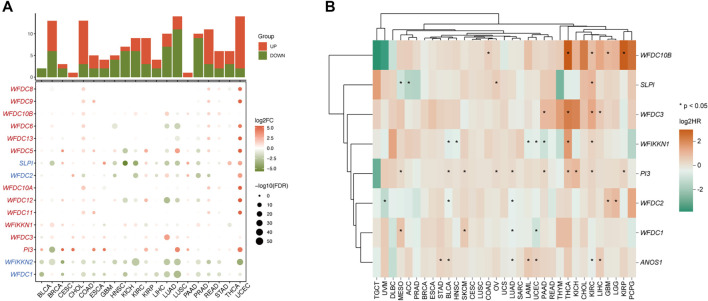
**Landscape of WFDC family genes expression and survival in pan-cancer.** (A) Bubble heatmap of WFDC family genes expression across tumor types, with the stacked bar chart above visualizing the number of WFDC family genes that are upregulated or downregulated in each cancer type. Genes with elevated expression in most tumors compared to normal tissue are labeled in red, while those with significantly reduced expression are labeled in blue. (B) Survival map based on univariate Cox regression analysis of WFDC family genes for OS across multiple cancers. *P < 0.05. FC: Fold change; FDR: False discovery rate; OS: Overall survival; HR: Hazard ratio; WFDC: Whey acidic protein four-disulfide core.

A schematic workflow of the experimental procedures is shown in Figure 1. Transcriptional profiles of WFDC genes across malignancies were systematically evaluated through a pan-cancer analysis of TCGA datasets. Distinct expression patterns emerged across cancer types (Figure 2A), with heatmap visualization highlighting pronounced upregulation of WFDC family genes in colon adenocarcinoma (COAD) and uterine corpus endometrial carcinoma (UCEC). In contrast, lung adenocarcinoma (LUAD), lung squamous cell carcinoma (LUSC), and prostate adenocarcinoma (PRAD) exhibited predominant transcriptional suppression. *SLPI*, *WFDC2*, *WFIKKN2*, and *WFDC1* consistently showed low expression across tumor types, whereas *WFDC5*, *WFDC3*, and *WFIKKN1* displayed heterogeneous expression profiles. Univariate Cox regression analysis was used to assess the prognostic relevance of WFDC genes across various cancers (Figure 2B). The resulting survival map revealed significant associations between specific WFDC members and OS in select malignancies. Notably, *WFDC10B*, *WFDC3*, *PI3*, and *ANOS1* were linked to poorer prognosis, as reflected by elevated hazard ratios. Conversely, *SLPI*, *WFIKKN1*, *WFDC2*, and *WFDC1* demonstrated variable prognostic effects across cancer types, suggesting context-dependent, microenvironment-driven functional duality. These trends were further supported by survival validation analyses using the GSCA database (Figure S1A and S1B). Bubble charts illustrated the associations of WFDC gene expression with multiple clinical outcomes, including OS, progression-free survival (PFS), disease-free interval (DFI), and DSS. Stage-specific transcriptional patterns were also characterized (Figure S1C), revealing distinct stage-dependent signatures among *WFDCs*. Longitudinal analysis of expression trends during tumor progression (Figure S1D) demonstrated dynamic modulation of WFDC gene expression across cancer stages. Collectively, these findings underscore the malignancy-specific transcriptional regulation of the WFDC family and highlight their potential roles in tumor progression and clinical outcomes.

### Expression and prognostic value of WFDC family in PAAD

**Figure 3.**
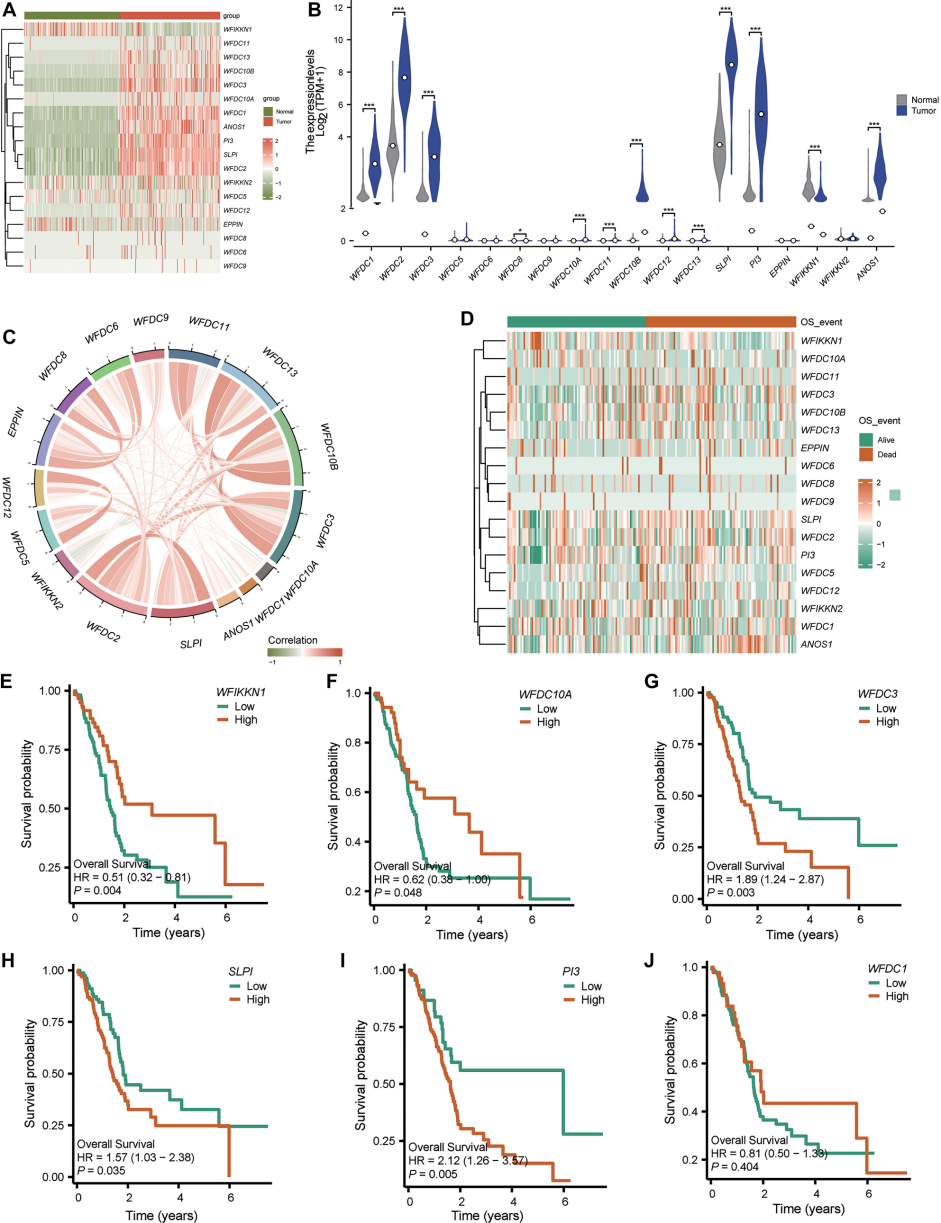
**The expression and prognostic value of WFDC family genes in pancreatic cancer.** (A) Heatmap; (B) Violin plots of the different mRNA expression of WFDC family genes between normal and tumor tissues in PAAD based on the TCGA and the GTEx datasets; (C) Chord diagram of Pearson correlations among WFDC family genes in PAAD; (D) Heatmap of WFDC family genes expression based on OS events; (E–J) KM curves of OS between patients with high and low WFDC family genes expression level. *P < 0.05, ***P < 0.001. OS: Overall survival; HR: Hazard ratio; KM: Kaplan–Meier; WFDC: Whey acidic protein four-disulfide core.

Comparative expression analysis of WFDC genes between PAAD tumors and normal pancreatic tissues was performed using integrated TCGA-GTEx datasets. Visualization of the transcriptional landscape revealed marked differences in WFDC gene expression profiles between malignant and non-malignant samples (Figure 3A). Statistical analysis showed significant tumor-specific upregulation of *WFDC1*, *WFDC2*, *WFDC3*, *SLPI*, *PI3*, and *ANOS1* (*P* < 0.0001), while *WFIKKN1* was notably downregulated in neoplastic tissues (*P* < 0.0001) (Figure 3B). Correlation analysis revealed complex interrelationships among WFDC genes in PAAD, with strongly correlated expression patterns among genes exhibiting similar regulation (Figure 3C). A comprehensive correlation matrix was also constructed to quantify these relationships (Figure S2), identifying several significant associations. Notably, *WFDC3* showed strong positive correlations with multiple family members, suggesting its potential central role in regulating WFDC family functions in PAAD. A detailed survival analysis—encompassing OS, DSS, and PFI—was conducted to assess the prognostic value of WFDC genes (Figure 3D; Figure S3). Initial OS-related associations were visualized using a heatmap (Figure 3D). *WFDC3* consistently demonstrated prognostic significance across multiple cancers, particularly in PAAD, showing associations with poorer OS, DSS, and PFI outcomes (Figure S4). Elevated *WFDC3* expression was associated with reduced OS (HR ═ 1.89, 95% CI ═ 1.24–2.87, *P* ═ 0.003; Figure 3G), DSS (HR ═ 2.02, 95% CI ═ 1.26–3.02, *P* ═ 0.003; Figure S3A), and PFI (HR ═ 1.64, 95% CI ═ 1.11–2.42, *P* ═ 0.014; Figure S3B). In contrast, *WFIKKN1* expression correlated with improved clinical outcomes. Kaplan–Meier survival curves revealed that higher *WFIKKN1* expression was significantly associated with longer OS (HR ═ 0.51, 95% CI ═ 0.32–0.81, *P* ═ 0.004; Figure 3E), DSS (HR ═ 0.56, 95% CI ═ 0.33–0.94, *P* ═ 0.0028; Figure S3A), and PFI (HR ═ 0.44, 95% CI ═ 0.24–0.73, *P* ═ 0.001; Figure S3B).

**Figure 4.**
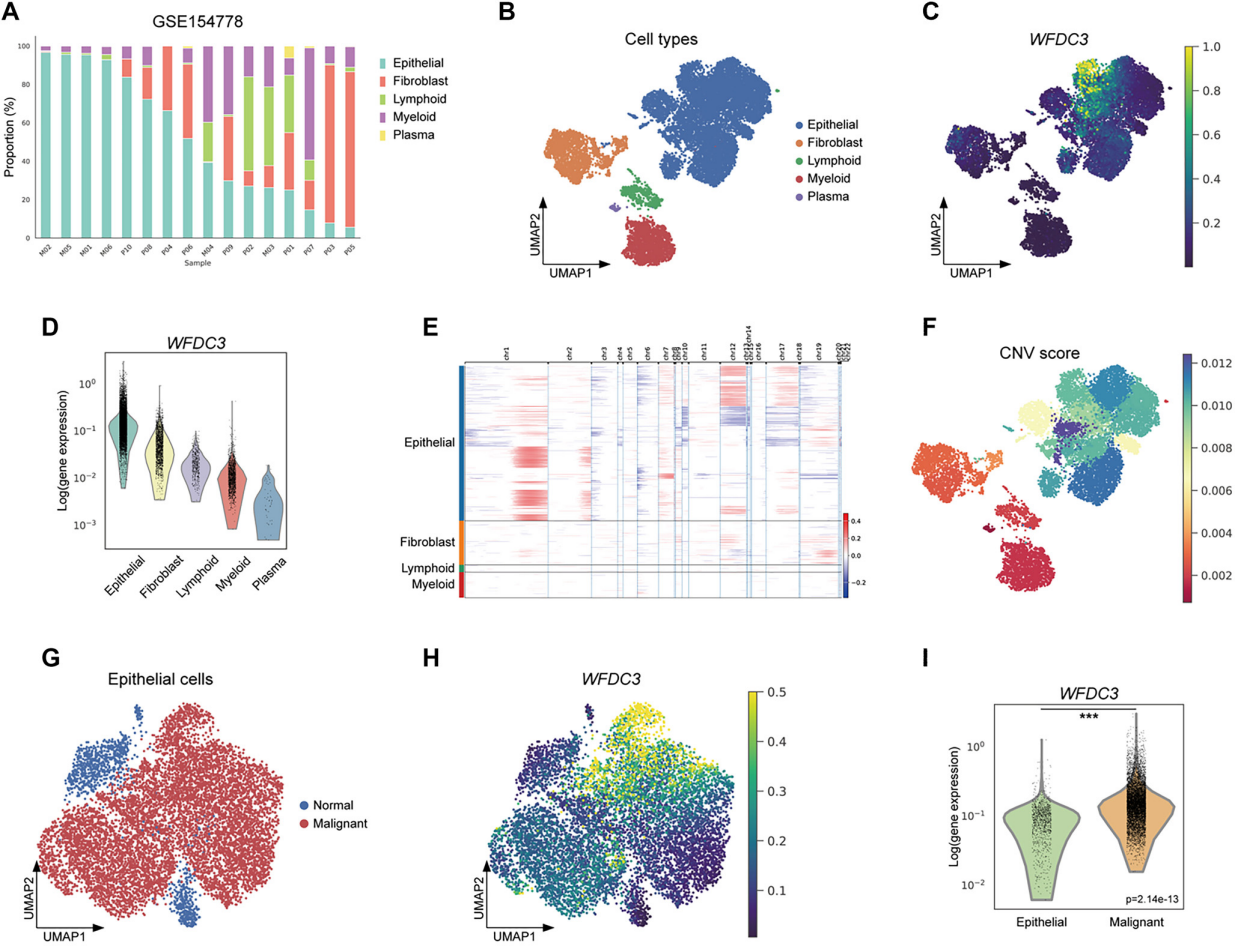
***WFDC3* exhibited specific expression in malignant epithelial cells of PAAD.** (A) The GSE154778 dataset comprises scRNA-seq data from 10 primary and six metastatic PAAD specimens, with cellular composition summarized across five major categories: Epithelial, fibroblast, lymphoid, myeloid, and plasma cells; (B) Major cell type distribution was visualized through UMAP dimensionality reduction; (C) Transcriptional profiles of *WFDC3* across all cellular subsets; (D) Violin plots depicted normalized *WFDC3* expression intensities within the five principal cell lineages; (E) InferCNV analysis was implemented using stromal cells as a reference population; (F) CNV scores were projected onto UMAP coordinates for spatial resolution; (G) Malignant epithelial cells were distinguished from normal counterparts based on elevated chromosomal instability; (H) UMAP visualization and (I) Violin plots compared *WFDC3* expression between malignant and non-malignant epithelial populations. Statistical significance was assessed via Mann–Whitney *U* testing (*P < 0.05, **P < 0.01, ***P < 0.0001). scRNA-seq: Single-cell RNA sequencing; WFDC: Whey Acidic protein four-disulfide core.

### *WFDC3* correlates with malignant evolution of ductal cells in PAAD

Transcriptional profiling using TCGA datasets revealed elevated *WFDC3* expression in PAAD tumor tissues compared to adjacent or normal pancreatic tissues (Figure S5). scRNA-seq data from TISCH showed that *WFDC3* is predominantly expressed in malignant epithelial cell clusters (Figure S6). The GSE154778 dataset includes scRNA-seq profiles from 10 primary and six metastatic PAAD specimens, summarizing the proportions of five major cell types: epithelial cells, fibroblasts, lymphoid cells, myeloid cells, and plasma cells (Figure 4A). UMAP dimensionality reduction effectively distinguished these cell populations, with epithelial cells forming the largest cluster (Figure 4B). Marker genes for each annotated cluster are shown in Figure S7. Notably, *WFDC3* exhibited strong cell-type specificity, with predominant expression in epithelial cells, as demonstrated by UMAP visualization (Figure 4C). Quantitative analysis confirmed significantly higher *WFDC3* expression in epithelial cells compared to fibroblasts, lymphoid cells, myeloid cells, and plasma cells (Figure 4D). InferCNV analysis, using stromal cells as a reference, revealed distinct chromosomal copy number variations (Figure 4E), which were mapped onto the UMAP to visualize CNV scores (Figure 4F). This approach enabled identification of malignant epithelial cells based on chromosomal instability patterns. Subsequent stratification of epithelial cells into normal and malignant subsets (Figure 4G) enabled detailed evaluation of *WFDC3* expression. UMAP visualization (Figure 4H) and statistical analysis (Figure 4I) showed significantly elevated *WFDC3* expression in malignant vs normal epithelial cells (*P* ═ 2.14e-13). Additional scRNA-seq analysis from our unpublished independent dataset further examined the cellular distribution and potential role of *WFDC3* in tumor progression. UMAP plots identified distinct cell populations (Figure S8A), with *WFDC3* expression mapped across them (Figure S8B). Detailed analysis indicated predominant *WFDC3* expression in ductal cells (Figure S8C), with significantly higher expression in IPMN and PDAC ductal cells compared to normal uninvolved (UNIN) ductal cells (Figure S8D and S8E, ***P < 0.001), supporting a role for *WFDC3* in the malignant transformation of ductal cells in PAAD. Clinicopathological correlations of *WFDC3* expression in PAAD were evaluated systematically (Figure S9A). Advanced T-stage tumors (T3&T4) exhibited significantly higher *WFDC3* expression than early-stage tumors (T1&T2; *P* < 0.05). Similarly, patients with residual tumors (R1&R2) had elevated *WFDC3* levels compared to those with complete resections (R0; *P* < 0.05). Although N1-stage tumors showed a trend toward increased *WFDC3* expression relative to N0-stage, this difference was not statistically significant. Genomic mutation analysis from the TCGA-PAAD cohort revealed distinct mutational landscapes between high and low *WFDC3* expression groups (Figure S9B and S9C). In the high-expression group, *KRAS* mutations were detected in 84 of 88 samples (95.45%), frequently accompanied by alterations in *TP53* (77%), *SMAD4* (25%), and *CDKN2A* (23%). Additional but less frequent mutations included *TTN* (17%), *RNF43* (9%), and *MUC16* (9%). The low-expression group also had *KRAS* as the most frequently mutated gene, though at a lower frequency (76.25%; 61 of 80 samples). This group maintained a similar driver gene profile with *TP53* (50%), *SMAD4* (22%), and *CDKN2A* (11%), albeit at reduced frequencies. These differences suggest potential molecular mechanisms linking *WFDC3* expression to pancreatic tumorigenesis and progression.

### Functional enrichment analysis of *WFDC3* related DEGs in PAAD

**Figure 5.**
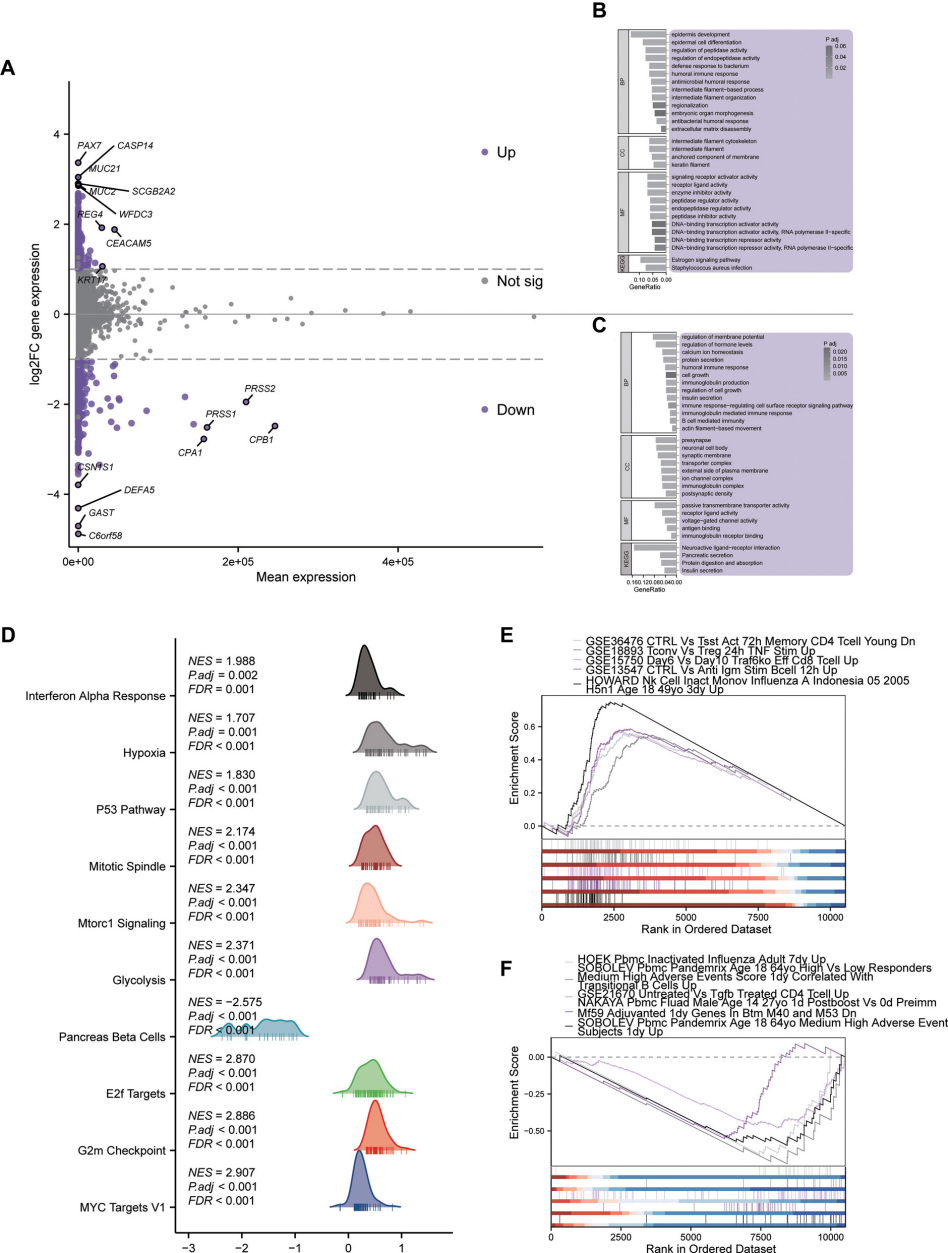
**Transcriptional landscape and pathway modulation across *WFDC3* expression strata.** (A) MA (M-vs-A) plot demonstrated genome-wide differential expression patterns; (B) Functional categorization of upregulated transcriptional variants in *WFDC3*-high cohorts through GO and KEGG pathway enrichment; (C) GO/KEGG profiling of downregulated genes in *WFDC3*-elevated specimens; (D–F) Gene set enrichment profiling (GSEA) of transcriptional variants across expression clusters. GSEA: Gene Set Enrichment Analysis; WFDC: Whey acidic protein four-disulfide core; KEGG: Kyoto Encyclopedia of Genes and Genomes; GO: Gene Ontology.

To elucidate *WFDC3’s* molecular mechanisms, systematic transcriptome profiling was performed. Comparative analysis between *WFDC3*-high and -low expression cohorts identified 3100 DEGs, including 820 upregulated and 2280 downregulated transcripts. An MA plot (Figure 5A) highlighted notable upregulation of *PAX7*, *CASP14*, and *MUC21*, alongside significant downregulation of *PRSS1*, *PRSS2*, *CPB1*, and *DEFA5*. GO analysis of upregulated DEGs revealed significant enrichment (*P* < 0.05) in biological processes, such as epidermal morphogenesis, protease activity modulation, and immune regulation (Figure 5B). Cellular component analysis indicated enrichment in intermediate filament networks, while molecular function analysis pointed to signaling receptor activation and transcriptional regulation. KEGG pathway analysis identified upregulation in estrogen signaling and Staphylococcus aureus infection pathways. In contrast, downregulated DEGs were enriched in pathways related to transmembrane potential homeostasis, hormonal regulation, and immune modulation (Figure 5C). These genes were associated with synaptic structures and ion channel complexes at the cellular level, and predominantly involved in transmembrane transport and receptor activities at the functional level. KEGG analysis further indicated significant downregulation in pancreatic secretion, insulin secretion, and protein digestion pathways. GSEA identified ten significantly altered pathways (FDR < 0.001) (Figure 5D). Among these, MYC targets V1 (NES ═ 2.907), G2M checkpoint (NES ═ 2.886), and E2F targets (NES ═ 2.870) showed strong positive enrichment, whereas the pancreatic beta cell pathway exhibited pronounced negative enrichment (NES ═ −2.575). Additional enrichment was observed in interferon alpha response, hypoxia, and the p53 pathway, indicating involvement of complex regulatory networks. Further GSEA (Figure 5E and 5F) uncovered distinct immunological signatures and metabolic alterations, suggesting comprehensive reprogramming of the immune microenvironment in response to *WFDC3* expression levels.

### Relationship between *WFDC3* expression and tumor-infiltrating immune cells in PAAD

**Figure 6.**
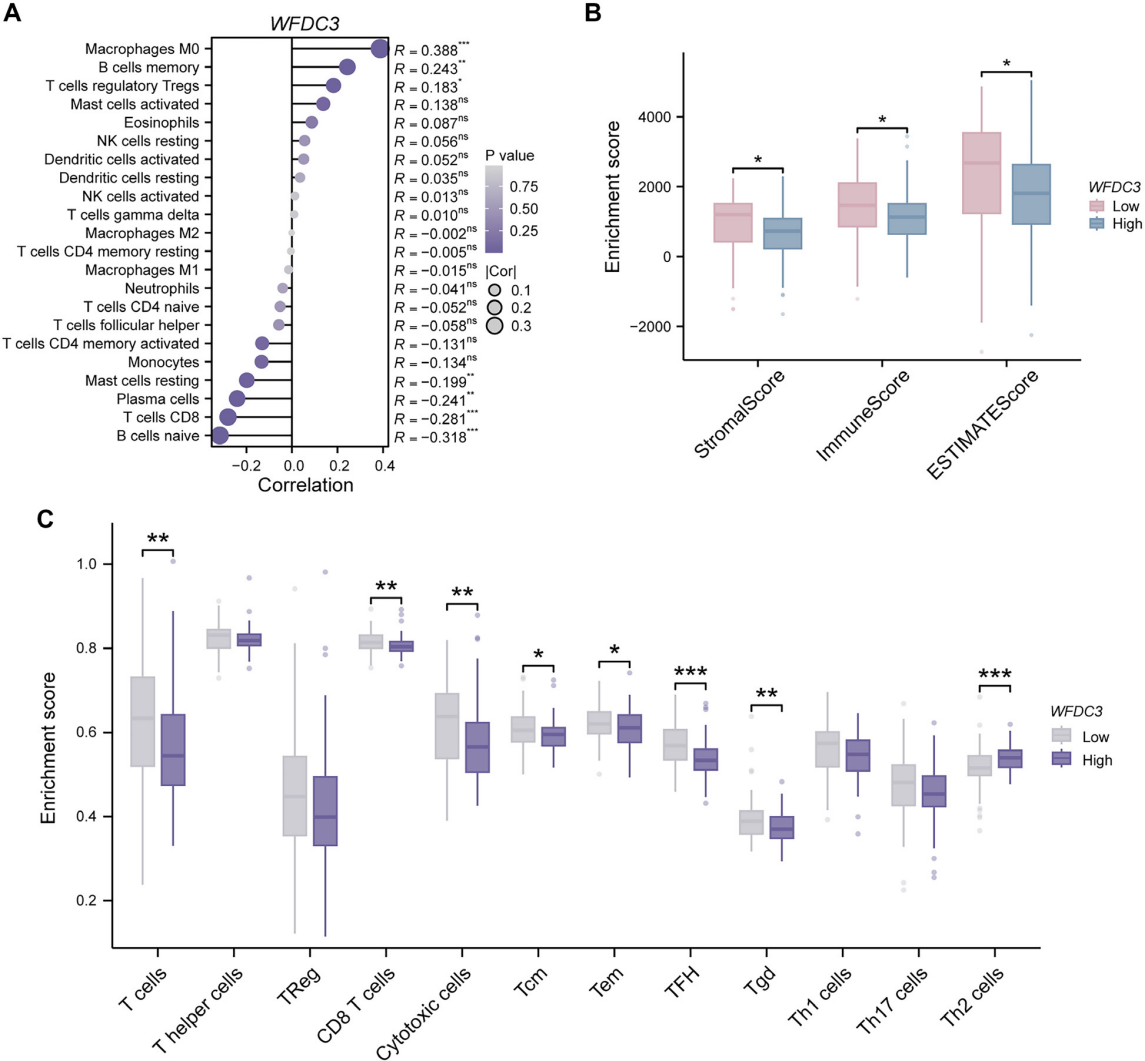
**Immune microenvironment characterization associated with *WFDC3* expression in TCGA-PAAD cohort.** (A) Immune infiltration patterns associated with *WFDC3* expression in PAAD were evaluated using CIBERSORT methodology; (B) Comparative assessment of stromal components, immune infiltration metrics, and composite ESTIMATE scores between *WFDC3* expression cohorts was performed via the ESTIMATE algorithm; (C) Quantitative profiling of principal T lymphocyte subsets across *WFDC3* expression strata was conducted through ssGSEA-based computational analysis. *P < 0.05, **P < 0.01, ***P < 0.001. GSEA: Gene Set Enrichment Analysis; WFDC: Whey acidic protein four-disulfide core.

Association studies between *WFDC3* expression and immune cell subsets across various malignancies revealed complex immunomodulatory networks (Figure S10). CIBERSORT deconvolution analysis identified distinct correlations between *WFDC3* levels and immune cell infiltration in PAAD (Figure 6A). Among these, M0 macrophages exhibited the strongest positive correlation (*R* ═ 0.388, *P* < 0.001), followed by memory B cells (*R* ═ 0.243, *P* < 0.01) and regulatory T cells (Tregs; *R* ═ 0.183, *P* < 0.05). In contrast, naïve B cells showed the most pronounced negative correlation (*R* ═ –0.318, *P* < 0.001), with CD8^+^ T lymphocytes (*R* ═ −0.281, *P* < 0.001) and plasma cells (*R* ═ −0.241, *P* < 0.01) also inversely associated with *WFDC3* expression. ESTIMATE-based computational analysis revealed significant differences in tumor microenvironment parameters between high and low *WFDC3* expression groups (Figure 6B). Stromal (*P* < 0.05), immune (*P* < 0.05), and composite ESTIMATE scores (*P* < 0.05) all varied significantly, underscoring *WFDC3’s* impact on the tumor microenvironment architecture. ssGSEA-based quantification of T cell subtypes further showed that *WFDC3*-high tumors had reduced infiltration of total T cells (*P* < 0.01), CD8^+^ T cells (*P* < 0.01), and cytotoxic subsets (*P* < 0.01) (Figure 6C). Decreased infiltration was also observed in central memory (Tcm), effector memory (Tem), follicular helper (TFH), and γ δ T cells (Tgd). Notably, T helper 2 cells (Th2) were paradoxically elevated in *WFDC3*-high tumors (*P* < 0.001), emphasizing the multifaceted immunoregulatory role of *WFDC3* in the pancreatic tumor microenvironment. Together, these findings suggest that *WFDC3* significantly influences the immune landscape in PAAD, potentially promoting immune evasion and tumor progression through modulation of specific immune cell populations.

### WFDC3 promoted PAAD cell migration and invasion *in vitro*

**Figure 7.**
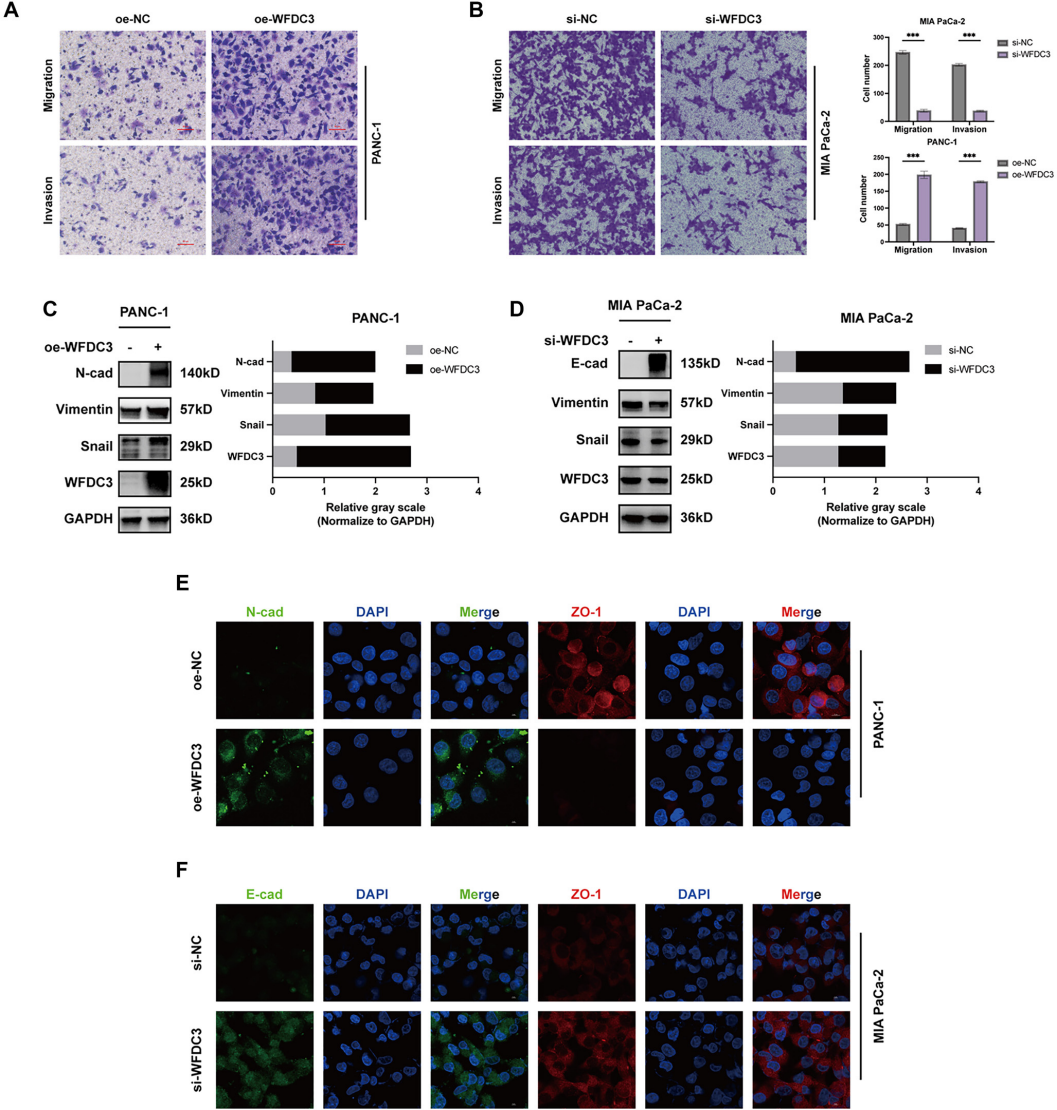
**WFDC3 promoted PAAD cell migration and invasion *in vitro*.** Transwell assays were used to evaluate the migration and invasion capacity of WFDC3 overexpression in PANC-1 (A) and WFDC3 knockdown in MIA PaCa-2 (B), scale bar: 50µm. (C and D) Western blot together with gray values and (E and F) Immunofluorescence staining was used to detect the expression of EMT related proteins, scale bar: 5µm. ***P < 0.001. EMT: Epithelial–mesenchymal transition; WFDC: Whey acidic protein four-disulfide core.

Transcriptomic profiling revealed elevated *WFDC3* expression in PAAD, prompting investigation into its regulatory mechanisms. Protein-level validation of WFDC3 expression was performed across normal pancreatic epithelial HPNE cells and several PAAD cell lines (MIA PaCa-2, PANC-1, AsPC-1, and BxPC-3) (Figure S11A). Comparative analysis confirmed consistent WFDC3 upregulation in malignant cell lines relative to HPNE controls, with the highest expression observed in MIA PaCa-2 and BxPC-3 cells. Gain-of-function studies were conducted by plasmid-mediated WFDC3 overexpression in PANC-1 cells, with successful transcriptional and translational upregulation confirmed by qRT-PCR (Figure S11B) and ELISA (Figure S11C). Cell proliferation dynamics were assessed using CCK-8 assays, colony formation assays, and EdU incorporation analyses. Longitudinal CCK-8 assays revealed no significant difference in viability between WFDC3-overexpressing and control cells (Figure S11D). This lack of proliferative effect was corroborated by comparable colony-forming abilities (Figure S11E) and similar proportions of EdU-positive cells (Figure S11F). Our previous studies demonstrated WFDC3-mediated suppression of epithelial–mesenchymal transition (EMT) in CRC [16]. In contrast, current findings indicate that WFDC3 promotes PAAD cell migration and invasion through EMT modulation (Figure 7). Transwell assays showed significantly increased migration and invasion in WFDC3-overexpressing PANC-1 cells (Figure 7A), while WFDC3 knockdown in MIA PaCa-2 cells reduced these capabilities (Figure 7B). These results establish WFDC3 as a facilitator of PAAD cell motility and invasiveness. To elucidate the underlying mechanisms, EMT marker expression was analyzed. Overexpression of WFDC3 in PANC-1 cells led to increased levels of mesenchymal markers, including N-cadherin, Vimentin, and Snail (Figure 7C), indicative of EMT induction. This was further supported by IF staining, which showed decreased expression of the tight junction protein ZO-1 and increased N-cadherin levels in WFDC3-overexpressing cells (Figure 7E). Conversely, WFDC3 knockdown in MIA PaCa-2 cells led to upregulation of the epithelial marker E-cadherin and downregulation of mesenchymal markers Vimentin and Snail (Figure 7D). Additionally, ZO-1 and E-cadherin expression were elevated following WFDC3 knockdown (Figure 7F). In summary, WFDC3 facilitates PAAD progression by enhancing migration and invasion through EMT induction. These findings position WFDC3 as a key regulator of pancreatic cancer aggressiveness and highlight its potential as a therapeutic target for metastasis suppression in PAAD.

**Figure 8.**
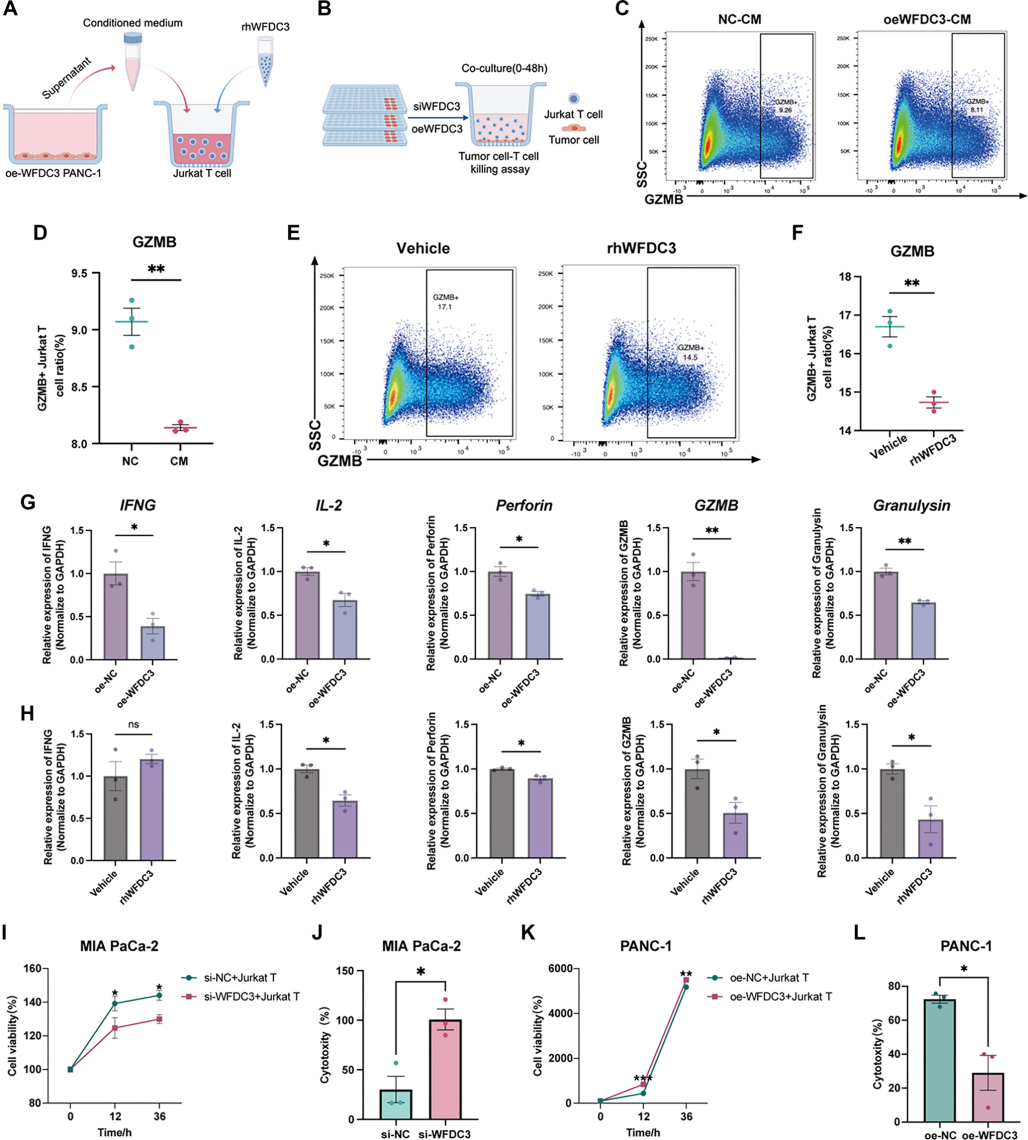
**WFDC3 inhibited T cell cytotoxicity in PAAD.** (A and B) Schematic representation of the experimental design by Figdraw. (C and D) Levels of GZMB in Jurkat T cells treated with conditional medium collected from WFDC3 overexpressed PANC-1 cells was assessed via flow cytometric analysis. (E and F) Levels of GZMB in Jurkat T cells treated with rhWFDC3 (10 ng/mL) was assessed via flow cytometric analysis. qRT-PCR was performed to evaluate T cell cytotoxicity-related markers using RNA retracted from PANC-1 cells after (G) transfected with WFDC3 overexpression plasmid or (H) treated with rhWFDC3 (10 ng/mL). (I) CCK-8 assay and (J) LDH release assay was performed in WFDC3-knockdown MIA PaCa-2 cells cocultured with Jurkat T cells to evaluate T cell cytotoxicity. (K) CCK-8 assay and (L) LDH release assay was performed in WFDC3-overexpression PANC-1 cells cocultured with Jurkat T cells. *P < 0.05, **P < 0.01, ***P < 0.001. WFDC: Whey acidic protein four-disulfide core; GZMB: Granzyme B; qRT-PCR: Quantitative real-time PCR.

### WFDC3 inhibited T cell cytotoxicity in PAAD

Functional enrichment analysis provided evidence that *WFDC3* exerts systemic immunosuppressive effects on T cells. To further evaluate its impact on T cell cytotoxicity, multiple experimental approaches were employed (Figure 8A and 8B). Flow cytometry revealed that treatment with recombinant human WFDC3 (rhWFDC3, 10 ng/mL) significantly reduced GZMB expression in Jurkat T cells compared to the vehicle control (*P* < 0.01) (Figure 8C and 8D). Similarly, conditioned medium from WFDC3-overexpressing PANC-1 cells (oeWFDC3-CM) markedly decreased the proportion of GZMB-positive Jurkat T cells relative to that cultured in normal control medium (NC-CM) (Figure 8E and 8F). Molecular analyses corroborated these findings, showing broad suppression of T cell cytotoxic function. qRT-PCR demonstrated that under WFDC3-overexpressing conditions, the expression of key cytotoxic mediators—including IFNG, IL2, Perforin, GZMB, and Granulysin (GNLY)—was significantly reduced (*P* < 0.05) (Figure 8G), with similar expression patterns observed following rhWFDC3 treatment (Figure 8H). Furthermore, co-culture experiments provided strong support for WFDC3’s immunosuppressive role. Co-culturing Jurkat T cells with WFDC3-knockdown MIA PaCa-2 cells enhanced T cell-mediated killing, as evidenced by decreased cell viability in CCK-8 assays (Figure 8I) and increased cytotoxicity in LDH release assays (Figure 8J). Conversely, PANC-1 cells overexpressing WFDC3 were more resistant to T cell-mediated killing, maintaining higher viability (Figure 8K) and exhibiting significantly reduced LDH release (*P* < 0.05) (Figure 8L). Collectively, these findings indicate that WFDC3 acts as a potent immunosuppressive factor in pancreatic cancer, impairing T cell cytotoxicity through both direct suppression of cytotoxic effector expression and reduced susceptibility to T cell-mediated cell killing.

### Establishment and validation of the prognostic model based on *WFDCs*

**Figure 9.**
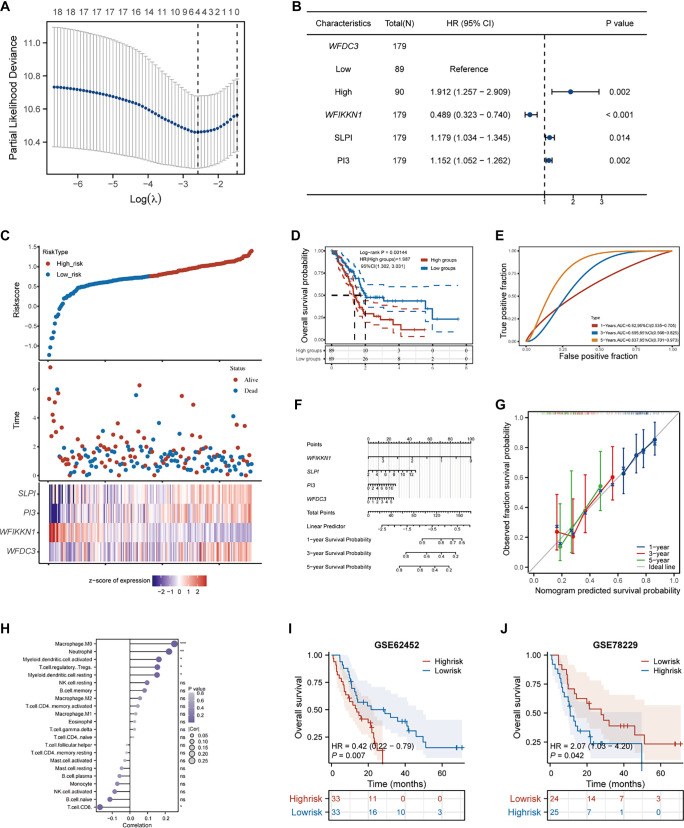
**Prognostic risk model derivation utilizing WFDC family genes in TCGA-PAAD cohort.** (A) LASSO regression analysis of 18 WFDC family members; (B) Univariate Cox regression forest plot identifying four OS-associated WFDC genes; (C) Patient stratification via median risk score thresholding (upper), with survival status distribution (middle) and four-gene expression patterns (lower) visualized; (D) Kaplan–Meier survival curves comparing high- vs low-risk cohorts; (E) ROC curve validation of risk score predictive accuracy; (F) Multivariate nomogram for pancreatic cancer survival probability estimation; (G) Calibration curves evaluating one-/three-/five-year OS prediction concordance; (H) Immune cell correlation profiling with risk model parameters. (I and J) The Kaplan–Meier analysis of the external validation cohorts. *P < 0.05, **P < 0.01, ***P < 0.001. WFDC: Whey acidic protein four-disulfide core; OS: Overall survival.

A prognostic model for PAAD based on WFDC family genes was developed and validated using the TCGA-PAAD cohort. LASSO regression analysis of 18 WFDC genes identified four key genes—*WFDC3*, *WFIKKN1*, *PI3*, and *SLPI*—that were significantly associated with OS (Figure 9A). Univariate Cox regression confirmed the prognostic relevance of these biomarkers: *WFIKKN1* was associated with a protective effect (HR < 1, *P* < 0.001), while *WFDC3*, *PI3*, and *SLPI* were linked to worse outcomes (HR > 1, *P* > 0.05) (Figure 9B). A multivariable risk model was constructed using the formula: Risk score ═ 0.068 × *WFDC3* + (−0.5398) × *WFIKKN1* + 0.041 × *PI3* + 0.0738 × *SLPI*. Patients were stratified into high- and low-risk groups based on the median risk score. Multimodal visualizations, including scatter plots and heatmaps, illustrated clear associations between gene expression patterns and survival, with elevated mortality observed in the high-risk group (Figure 9C). Kaplan–Meier survival curves confirmed a significant difference in OS between risk groups, with higher mortality in the high-risk cohort (*P* ═ 0.00144) (Figure 9D). Model performance was evaluated via time-dependent ROC analysis, yielding AUC values of 0.62 (one-year), 0.695 (three-year), and 0.837 (five-year), supporting the model’s predictive validity (Figure 9E). A multivariate nomogram incorporating the four-gene signature was created to estimate one-, three-, and five-year survival probabilities, providing a practical tool for individualized risk assessment (Figure 9F). Calibration curves showed strong agreement between predicted and observed outcomes across all time points, underscoring the model’s accuracy (Figure 9G). Correlation analysis between risk scores and immune infiltration revealed significant associations with various immune cell types, including macrophages and T-cell subsets, suggesting the model reflects key interactions between tumor progression and the immune microenvironment (Figure 9H). To validate the model externally, two independent PAAD cohorts from the GEO database (GSE62452 and GSE78229) were analyzed. Patient risk scores were calculated using the established formula, and stratification by median values effectively divided them into high- and low-risk groups. Kaplan–Meier analysis confirmed significantly better survival in the low-risk groups across both datasets (*P* < 0.05, Figure 9I and 9J), reinforcing the model’s generalizability and prognostic utility. These findings support the use of the four-gene *WFDCs*-based signature for risk stratification and potentially guiding immunotherapeutic strategies in PAAD.

## Discussion

The WFDC family members are multifaceted contributors to oncological processes, modulating inflammation, immune regulation, cell adhesion, signaling pathways, and extracellular matrix remodeling. Collectively, these functions promote tumorigenesis and disease progression, positioning the WFDC family as a critical research frontier in cancer biology [25, 26]. Although functional conservation exists among family members, notable differences in expression patterns and mechanisms are observed. For example, WFDC1 is downregulated in the stroma of prostate tumors, regulates the COX-2 pathway, and mediates immune cell interactions—suggesting a tumor-suppressive role [27–30]. In contrast, WFDC4 promotes metastasis in ovarian cancer via PI3K-AKT pathway activation and MMP-9 secretion [31]. WFDC2, one of the most extensively studied members, displays a functional dichotomy: it serves as a diagnostic biomarker in lung, ovarian, and prostate cancers [11, 15, 32, 33], with clinical utility as a serum marker for ovarian cancer [34], yet paradoxically suppresses prostate cancer metastasis by inhibiting EGFR-mediated cell migration [15]. These context-dependent roles highlight the need for systematic pan-cancer studies to clarify the tissue-specific oncogenic and tumor-suppressive functions of WFDC proteins. To explore the regulatory mechanisms of WFDC family members across cancers, a comprehensive analytical framework was developed. Pan-cancer transcriptional profiling revealed heterogeneous *WFDCs* expression, indicative of functional plasticity dependent on tumor context. Univariate Cox survival modeling identified significant correlations between *WFDCs* expression and OS in multiple cancers, with high expression levels often associated with poorer prognosis. PAAD, the third leading cause of cancer-related death globally, exhibits a 10% five-year survival rate and a median survival of less than six months [2, 35]. Limited treatment options, resistance to therapy, and a lack of effective biomarkers contribute to its poor prognosis, underscoring the urgent need for novel therapeutic strategies [1]. However, the role of WFDC proteins in PAAD remains largely unexplored. To address this gap, we conducted an in-depth analysis of WFDC family members in PAAD using integrated TCGA and GTEx datasets. This enabled a comparison of mRNA expression between tumor and adjacent normal tissues, revealing that more than half of the WFDC genes were transcriptionally upregulated in tumors. These findings were validated across datasets, indicating conserved expression patterns. Correlation analyses—employing two complementary methods—revealed strong co-expression among WFDC members, suggesting potential synergistic roles in PAAD biology. Subsequent survival analyses identified WFDC3 and WFIKKN1 as both prognostic biomarkers and potential functional contributors to PAAD progression. Their consistent association with patient outcomes across multiple survival metrics underscores their clinical relevance. Furthermore, univariate Cox regression followed by LASSO modeling identified four key prognostic WFDC members (*WFDC3*, *WFIKKN1*, *SLPI*, and *PI3*). The resulting predictive model demonstrated strong performance in forecasting PAAD patient outcomes, supporting the translational potential of *WFDCs*-based prognostic tools.

Current research on *WFDC3* remains limited. A recent study investigating the functional roles of WFDC genes in various aspects of male fertility reported that *WFDC3* knockout mice did not exhibit any apparent reproductive defects [36]. Additionally, multi-omic analyses of PAAD conducted by Cao et al. [37] revealed upregulation of *WFDC3* at the transcriptomic level. In the present study, *WFDC3* was found to be significantly upregulated in PAAD and associated with poor prognosis—a finding that contrasts with previous reports of its tumor-suppressive role in CRC [16]. These results suggest that WFDC3 may exhibit complex, tissue-specific functions in the development of PAAD. Consistent with bulk RNA sequencing data, scRNA-seq analysis provided critical insights into the cellular distribution of *WFDC3*, confirming its predominant expression in malignant epithelial cells. Furthermore, a progressive increase in *WFDC3* mRNA levels from UNIN to IPMN to PDAC suggests its potential involvement in the malignant transformation of pancreatic ductal cells. This finding is particularly relevant given the ongoing challenges in elucidating the molecular mechanisms underlying PAAD oncogenesis. The principal oncogenic drivers in PAAD pathogenesis include *KRAS*, *CDKN2A*, *TP53*, and *SMAD4*. While *KRAS* and *CDKN2A* mutations typically arise during tumor initiation, aberrations in *TP53* and *SMAD4* are associated with disease progression and malignant transformation [38]. Our analysis revealed a correlation between *WFDC3* expression and mutations in these driver genes, with notably higher *KRAS* mutation frequencies observed in *WFDC*3-high cohorts. Clinically, advanced-stage PAAD patients exhibited significantly elevated *WFDC3* expression compared to early-stage patients. These findings support the role of *WFDC3* as a tumor-promoting factor and an independent adverse prognostic biomarker in PAAD. To elucidate the functional implications of *WFDC3*, we performed enrichment analyses on DEGs between *WFDC3*-high and -low expression groups. GO and KEGG pathway analyses revealed marked enrichment in cytoskeletal reorganization processes, particularly intermediate filament assembly and actin-mediated motility, indicating a potential role for *WFDC3* in modulating cellular structural plasticity. Substantial evidence links dynamic cytoskeletal remodeling to tumor metastasis [39, 40], suggesting that *WFDC3* may contribute to metastatic progression by influencing cytoskeletal dynamics. In addition, functional annotations highlighted significant involvement in immune regulatory pathways, including humoral immunity, B cell activation, and immunoglobulin-mediated responses. GSEA corroborated these findings and uncovered further associations with cell cycle regulation, notably G2/M checkpoint control and mitotic spindle organization. Immune-related pathways were particularly enriched, with the interferon-alpha response prominently represented. Further GSEA analyses revealed strong associations with T cell inhibition and broader modulation of immune responses. Enrichment scores varied notably across different immune cell states, underscoring *WFDC3’*s role in fine-tuning specific immune functions. These molecular insights highlight the dual regulatory role of *WFDC3* in cytoskeletal organization and immune response modulation. The enriched pathways align with prior observations linking *WFDC3* to tumor progression and survival, suggesting it may influence PAAD development through coordinated regulation of cell motility and immune dynamics.

Immune-checkpoint inhibitors (ICIs) have revolutionized cancer therapeutics through their novel mechanism of action [41, 42]. These agents enhance tumor targeting by promoting immune recognition of tumor-specific neoantigens, which are processed into immunogenic peptides that activate cytotoxic CD8^+^ T lymphocytes capable of eliminating malignant cells [43]. However, PAAD exhibits intrinsic resistance to ICIs, primarily due to two factors: a relatively low neoantigen burden compared to other malignancies and the presence of a complex, immunosuppressive TME. Together, these factors impair T cell activation and dampen antitumor immune responses [44–47]. Developing innovative strategies to overcome these immunosuppressive mechanisms remains an urgent research priority. PAAD is characterized by a highly reactive and desmoplastic stroma [48]. Investigating the interaction between cancer cells with upregulated *WFDC3* and other cellular components of the tumor immune microenvironment may yield novel insights into therapeutic strategies targeting *WFDC3*-mediated immune regulation. Our analysis revealed a negative association between *WFDC3* expression and multiple immune cell populations, suggesting broad immunosuppressive effects. Subsequent ESTIMATE analysis demonstrated that high *WFDC3* expression correlated with significantly lower ImmuneScore, StromaScore, and ESTIMATEScore, indicating reduced immune cell infiltration in the tumor stroma and impaired antitumor immune responses. Notably, *WFDC3* showed a strong negative correlation with various T cell subsets. Further analysis confirmed that elevated *WFDC3* expression was significantly associated with reduced infiltration and activity of multiple functional T cell subtypes, including cytotoxic T cells, CD8^+^ T cells, and T follicular helper cells—populations essential for sustaining antitumor immunity [49]. These findings suggest that *WFDC3* may contribute to immune evasion by suppressing T cell–mediated immune responses. Given its immunosuppressive role and negative association with T cell infiltration, we hypothesized that *WFDC3* expression might influence response to immunotherapy. However, due to the limited availability of immunotherapy-treated PAAD cohorts, we utilized a CRC immunotherapy dataset (GSE53127) for preliminary evaluation [50, 51]. The analysis revealed significantly higher *WFDC3* expression in responders compared to non-responders (Figure S12). This observation aligns with previous findings identifying *WFDC3* as a tumor suppressor in CRC and supports its potential role as a context-dependent modulator of immune sensitivity [16]. These findings underscore the need for further investigation into the predictive value of *WFDC3* in immunotherapy-treated cohorts, particularly in immune-refractory malignancies such as PAAD.

Building on previous research [16, 17] and the bioinformatic findings presented above, we hypothesized that WFDC3 functions as a dispensable regulator within the molecular modulation network of pancreatic cancer—a notion further substantiated by *in vitro* experiments. Elevated WFDC3 protein expression in pancreatic cancer cells was confirmed via Western blot analysis. Notably, gain- and loss-of-function experiments revealed that WFDC3 does not influence cell proliferation. This finding partially contradicts earlier reports, potentially due to the complex regulatory mechanisms in physiological environments, where multiple signaling pathways and cellular interactions collectively fine-tune WFDC3’s functional output. Nonetheless, we observed that WFDC3 promote PAAD cell migration and invasion through EMT induction, consistent with our analyses. The conserved structural domains of the WFDC family confer intrinsic protease inhibitory activity [52, 53], and growing interest surrounds their potential role in tumor immune regulation [54]. SLPI, for instance, has been shown to mediate its antiprotease-related immunoregulatory effects through various mechanisms [55, 56]. This study provides the first experimental evidence that WFDC3 exerts an immunosuppressive effect by attenuating T cell cytotoxicity, although the precise regulatory mechanisms remain unclear and warrant further investigation. While this study offers novel insights into the prognostic relevance of *WFDC3* in PAAD, several methodological limitations should be acknowledged. The use of publicly available datasets introduces potential selection bias, underscoring the need for validation in independent, large-scale PAAD cohorts with detailed clinicopathological annotations. Moreover, the clinical utility of WFDC3 as a biomarker, along with our prognostic model, requires thorough evaluation. Although *in vitro* experiments confirmed WFDC3’s pro-metastatic and immunosuppressive properties, systematic *in vivo* studies are essential to uncover the underlying mechanisms and assess the therapeutic potential of targeting WFDC3 in immunotherapy-resistant PAAD patients.

## Conclusion

In conclusion, our pan-cancer analytical framework revealed distinct expression patterns of the WFDC family and identified WFDC3 as a promising prognostic biomarker and therapeutic target in PAAD, owing to its dual association with metastatic progression and modulation of the immune microenvironment. This study highlights WFDC3’s critical regulatory role in PAAD pathogenesis and underscores the need for further mechanistic investigations to clarify its specific oncogenic pathways.

## Supplemental data

Supplemental data are available at the following link: https://www.bjbms.org/ojs/index.php/bjbms/article/view/12444/3874.

## Data Availability

The data supporting the findings of this study are available from the corresponding authors upon reasonable request.
